# Knockdown of subunit 3 of the COP9 signalosome inhibits C2C12 myoblast differentiation via NF-KappaB signaling pathway

**DOI:** 10.1186/s40360-017-0154-5

**Published:** 2017-06-17

**Authors:** Mariam A. Ba, Jeffrey Surina, Cherie A. Singer, Maria L. Valencik

**Affiliations:** Department of Pharmacology, University of Nevada School of Medicine, Reno, NV USA

**Keywords:** COP9 signalosome, CSN3, Differentiation, C2C12 myoblast, RNAi, NF-κB

## Abstract

**Background:**

The COP9 signalosome (CSN) is a conserved protein complex composed of 8 subunits designated CSN1-CSN8. CSN3 represents the third subunit of the CSN and maintains the integrity of the complex. CSN3 binds to the striated muscle-specific β1D integrin tail, and its subcellular localization is altered in differentiated skeletal muscle cells. However, the role of CSN3 in skeletal muscle differentiation is unknown. The main goal of this study was to identify whether CSN3 participates in myoblast differentiation and the signalling mechanisms involved using C2C12 cells as a skeletal muscle cell model.

**Methods:**

Small-hairpin (shRNA) was used to knockdown CSN3 in C2C12 cells. Differentiation was evaluated by immunostaining and confocal microscopy. Markers of differentiation, NF-κB signaling and CSN subunits expression, were assessed by immunoblotting and/or immunostaining. Cell proliferation was analysed by cell counting, flow cytometry and a 3-(4,5-dimethylthiazol-2-yl)-2,5-diphenyltetrazolium bromide (MTT) assay. Data were analyzed by one or two-way analysis of variance (ANOVA) followed by post-hoc testing.

**Results:**

Transduction of C2C12 cells with two distinct CSN3 shRNAs led to the production of two cells lines expressing 7% of CSN3 protein (shCSN3-Low) and 43% of CSN3 protein (CSN3-Med) compared to controls. Knockdown of CSN3 was accompanied by destabilization of several CSN subunits and increased nuclear NF-κB localization. shCSN3-Med cells expressed less myogenin and formed shorter and thinner myotubes. In contrast, the shCSN3-Low cells expressed higher levels of myogenin prior and during the differentiation and remained mononucleated throughout the differentiation period. Both CSN3 knockdown cell lines failed to express sarcomeric myosin heavy chain (MHC) protein during differentiation. The fusion index was significantly higher in control cells than in shCSN3-Med cells, whereas shCSN3-Low cells showed no cell fusion. Interestingly, CSN3 knockdown cells exhibited a significantly slower growth rate relative to the control cells. Cell cycle analysis revealed that CSN3 knockdowns delayed in S phase and had increased levels of nuclear p21/Cip1 and p27/Kip1.

**Conclusions:**

This study clarifies the first step toward unrevealing the CSN3/CSN-mediated pathways that controls C2C12 differentiation and proliferation. Further in vivo characterization of CSN/CSN3 may lead to the discovery of novel therapeutic target of skeletal muscle diseases such as muscular dystrophies.

## Background

The COP9 signalosome (CSN) is a highly conserved protein complex, consisting of 8 subunits designated CSN1-CSN8 in higher eukaryotes [1–3]. Six subunits (CSN1, −2, −3, −4, −7 and −8) contain a proteasome, CSN, and initiation factor 3 (PCI) domains, while two others (CSN5 and CSN6) have an Mpr1-Pad1-N-terminal (MPN) domain [4, 5]. The PCI and MPN domains are involved in CSN subunit-subunit interactions and deneddylation, respectively [6, 7]. In mammals, CSN participates in many cellular processes, including signal transduction [8], protein stability, protein phosphorylation [9–11], cell cycle regulation [12, 13] and apoptosis [14, 15]. The most characterized role of CSN is the regulation of protein degradation via ubiquitination and the degradation of polyubiquinated proteins by the 26S proteasome, a highly conserved protein involved in the degradation of polyubiquitinated proteins. CSN and the 26S proteasome lid share homology in their subunit sequence and composition, indicating that these two complexes could originate from a common ancestor [3, 16]. CSN3 is the third subunit of the CSN complex, and is highly expressed in the heart, skeletal muscle, brain, liver, kidney and testis [17, 18]. CSN3 maintains the integrity of the CSN holo-complex and is crucial for early mouse embryonic development [17]. Moreover, Hunter et al., 2009 [19] have shown altered CSN3 subcellular localization following skeletal muscle cell differentiation. This suggests that CSN3 potentially has a role in the differentiation process. Skeletal muscle differentiation is a multistep process characterized by the expression of early myogenic markers such as myogenin and cell cycle withdrawal followed by expression of muscle specific genes such as myosin heavy chain and actinin, culminating in myoblast-myoblast fusion into multinucleated elongated myotubes [20, 21]. This sequence of events is regulated by extracellular matrix (ECM) proteins and signaling events [22]. The ECM is a dynamic substrate that serves as a scaffold for cells, maintains tissue integrity and compartmentalization, and mediates cellular communications [23]. ECM proteins such as laminin, fibronectin and collagen, comprise the ECM scaffold and basement membrane, which is a thin sheet that surrounds cells [24, 25]. Laminin, when used as the ECM on tissue culture plates, enhances myoblast proliferation, migration, and differentiation more efficiently than fibronectin or collagen [26–28].

This study addressed the hypothesis that CSN3 mediates skeletal muscle differentiation in C2C12 cells, which are a well-established primary murine myoblast model cell line consistently used to study skeletal muscle differentiation in vitro [29]. To test this hypothesis, we generated C2C12 stable CSN3 knockdown cell lines via RNAi, and investigated the effects of CSN3 knockdown on CSN complex integrity and C2C12 proliferation and differentiation. Further, we determined the signaling pathway by which CSN3 modulates C2C12 differentiation to begin to identify targets for further study of potential therapeutic development.

## Methods

### Cell culture reagents

Growth media (GM) was composed of Dulbecco’s Modified Eagle Medium (DMEM) high glucose, 10% fetal bovine serum (FBS) +/− antibiotics (50 U/ml penicillin and 50 μg/ml streptomycin). Differentiation media (DM) contained DMEM, 2% horse serum and antibiotics. Serum free media comprised DMEM/F12 (1:1) supplemented with 1% insulin-transferrin-selenium (ITS) (BD Biosciences, Bedford, MA). DMEM high glucose, DMEM/F12, penicillin/streptomycin, FBS, trypsin, laminin, a 3-(4,5-dimethylthiazol-2-yl)-2,5-diphenyltetrazolium bromide (MTT) cell proliferation assay kit and heat inactivated horse serum were purchased from Invitrogen (Carlsbad, CA). Lipopolysaccharide (LPS), puromycin and lentiviral particles were obtained from Sigma (St. Louis, MO).

### Antibodies

Rabbit anti-CSN1, −CSN2 and –CSN8 were purchased from ENZO life Sciences (Farmingdale, NY). Rabbit anti-CSN3 and mouse anti-CSN5 (Jab1, ab495) were obtained from Bethyl Laboratories (Montgomery, TX) and Abcam (Cambridge, MA) respectively. Mouse anti-myogenin was purchased from BD Biosciences (San Jose, CA). Bizbenzimide and mouse anti-sarcomeric alpha actinin were purchased from Sigma (St. Louis, MO). Mouse anti-myosin heavy chain-clone MF-20 was obtained from Hybridoma Bank (Iowa City, Iowa). The mouse anti-tubulin, mouse anti-glyceraldehyde 3-phosphate dehydrogenase (GAPDH) and NF-κB antibodies were from Santa Cruz Biotechnologies (Santa Cruz, CA), and p21, p26 and CD6 from Cell Signaling Technology (Danvers, MA). Secondary antibodies used in immunoblotting were from LI-COR Biosciences (USA), and those used in immunostaining were from Invitrogen Molecular Probes (Eugene, OR).

### Cell culture and shRNA interference

Low passage C2C12 (ATCC CRL-1772, Manassas, VA) cells were maintained in GM + antibiotics at 37 °C in a humidified incubator with 5% CO_2_. One day before lentiviral infection, C2C12 were seeded at a density of 105 cells/cm^2^. On the day of infection, cells were fed with fresh GM lacking antibiotics, but containing 8 μg/ml polybrene (Sigma, St. Louis, MO). Following 5 min incubation, lentiviral particles encoding 5 distinct shRNAs targeting the CSN3 gene (shCSN3-89, 90, 91, 92 or 93) or a non-target shRNA control (shNT) were added to each well at a multiplicity of infection (MOI) of 30. This MOI led to optimal degree of knockdown without any toxicity and was chosen based on a prior time-course experiment. The plate was rocked every 10 min for the first hour. The next day, supernatant was removed and fresh GM was added. Stable cell lines were obtained by selecting puromycin (1.5 μg/ml) resistant cells for 10 days. Upon confluency, cells were subcultured and maintained in GM + antibiotics + puromycin for all subsequent experiments.

### Induction of differentiation

Myoblasts were seeded on laminin (10 μg/ml) coated plates and cultured until they reached 70–80% confluency. To induce differentiation, GM was replaced with DM. Cells were allowed to differentiate for 0 to 9 days and DM was replenished every 48 h. Differentiating cells were photographed with a Spot camera (Diagnostic instruments, Sterling Heights, MI) throughout the time course. Proteins were extracted from cells after 0, 1, 3, 5, 7 or 9 days of differentiation for immunoblotting analysis, and cells designated for immunostaining were fixed 5 days post-differentiation.

### Cell fractionation

Cells were washed twice in 1X phosphate buffered saline (PBS) containing 137 mM NaCl, 2.7 mM KCl, 4.3 mM Na_2_HPO4 and 1.4 mM KH_2_PO4, treated with 0.5 mL hypotonic buffer A (10 mM HEPES pH 7.9, 10 mM KCl, 0.1 mM EDTA) for 1 min and lysates transferred to 1.5 microcentrifuge tubes. Cells lysates were then incubated on ice for 15 min and centrifuged at 4 °C at 3000 rpm for 7 min. The supernatant (cytosolic fraction) was transferred in a new tube and pellet (nuclei) was subjected to a hypertonic buffer B treatment (20 mM HEPES, pH 7.9, 0.4 M NaCl, 1 mM EDTA) for 45 min. The nuclear fraction was collected by centrifugation at 13,000 rpm for 15 min. Both buffers A and B were supplemented with proteases inhibitors: 1 mM DTT, 0.5 mM PMSF, 5 μl of 10 μg/μl of aprotinin, leupeptin, and pepstatin A to 5 ml of buffer and 2% glycerol.

### Total cell protein extraction

Cells were washed twice in ice-cold 1X PBS, scraped into TNET (50 mM Tris, 300 mM NaCl, 5 mM EDTA and 1% Triton X-100) buffer supplemented with protease inhibitor cocktail (Roche, Indianapolis, IN),10 mM sodium fluoride and 1 mM sodium orthovanadate (Sigma, St. Louis, MO). Protein extracts were sonicated (Branson sonicator 450) for 5 s, centrifuged at 13,000 rpm for 10 min at 4 °C, and concentrations determined using bicinchoninic acid (BCA) assay (Pierce, Rockford, IL).

### Immunoblot analysis

Equal protein amounts of total protein (15–20 μg) were separated on 4–12% NuPAGE Bis-Tris gels (Invitrogen, Carlsbad, CA), and transferred to Hybond-C nitrocellulose membranes (Amersham Biosciences, UK). Blots were blocked for an hour then probed with the appropriate primary antibody. Blots were washed 4 × 10 min with 1X PBS plus 0.1% Tween-20. Secondary antibodies were applied for 1 h and blots washed as above, and scanned with LI-COR Odyssey Infrared Imaging System (LI-COR Biosciences). Immunoreactive bands were quantified by densitometry using LI-COR software (Odyssey 2.0). GAPDH or tubulin was used as an internal control on the same gels used to visual experimental antibodies.

### Immunostaining

Cells were fixed in 3.7% formaldehyde (Fisher, USA) for 10 min, permeabilized with 0.3% Triton X-100 (Fisher, USA) for 5 min and blocked in 2% bovine serum albumin (BSA, Roche, Indianapolis, IN) for 2 h. Cells were incubated overnight at 4 °C with mouse anti-sarcomeric α actinin. Samples were washed in 1X PBS plus 0.1% Tween-20 and incubated with goat anti-mouse Alexa 546 secondary antibody and bisbenzimide (nuclear stain) for 1 h at room temperature. All antibodies were diluted at 1:500 in 1% BSA containing 0.1% Tween-20. The fluorescent images were captured with a confocal microscope (Olympus FluoView 1000).

### Fusion index

To measure the fusion index, differentiated cells were stained with sarcomeric α actinin and imaged by confocal microscopy (Olympus FluoView 1000) using a 40X objective. Actinin-positive cells with at least three nuclei were considered as myotubes. The fusion index was determined as the percentage of nuclei in myotubes divided by the total number of nuclei per a given field.

### Cell proliferation assays

Cells were seeded at the same density on laminin (10 μg/ml) coated dishes, fed with GM overnight and then serum-starved. After 48 h growth arrest, the serum-free media was replaced with GM and cell proliferation was determined from 0 to 6 days by three independent methods. After trypsinization, cells were resuspended in equal volumes of 1X PBS and quantitated with a Coulter counter (Beckman Coulter Z series, Luton, Beds England). Alternatively, for flow cytometry analysis, cells were resuspended in 500 μl of 0.1% Tween 20, 2% formalin, 2.5 mM EDTA, 50 μg/ml propidium iodide (PI) and 84.5 μl beads/μl in PBS. The nuclei were counted with a LSR II flow cytometer (Becton Dickenson, San Jose, CA) using a known number of beads. The results were analyzed by FlowJo 8.8.7 (Ashland, OR). For MTT assays, GM media was removed 24 or 48 h post proliferation, cells were rinsed with 1X PBS and fed with 100 μl fresh medium (DMEM free of phenol red plus 10% FBS). MTT (10 μl of 5 mg/ml) was added to each well and incubated for 4 h and then dissolved in 100 μl of 100 mg/ml SDS-HCl during a 5 h incubation. All incubations were done at 37 °C in a humidified incubator and absorbance read at 560 nm.

### Cell cycle analysis by flow cytometry

The three stable cells lines were seeded at equal densities, growth arrested for 48 h and treated with GM containing 10% FBS. Cells were grown for 48 h and fixed in 70% ethanol overnight at −20 °C. The next day, cells were pelleted (1200 rpm for 10 min at 4 °C), washed in cold 1X PBS and stained with PI solution (50 μg/ml of PI and 100 μg/ml of RNAse A in PBS) for 30 min at 37 °C. The cell cycle was analyzed by flow cytometry immediately after PI staining. To discriminate doublets, the area versus height was assessed using FlowJo 8.8.7 (Ashland, OR).

### Statistics

Data were analyzed by one or two-way analysis of variance (ANOVA) when comparing multiple groups. Post-hoc analysis was performed with Turkey’s multiple comparison (one-way ANOVA) or Bonferroni (two-way ANOVA) tests. *P* ≤0.05 was considered statistically significant.

## Results

### Generation of CSN3 stable knockdowns in C2C12 cells

**Fig. 1:**
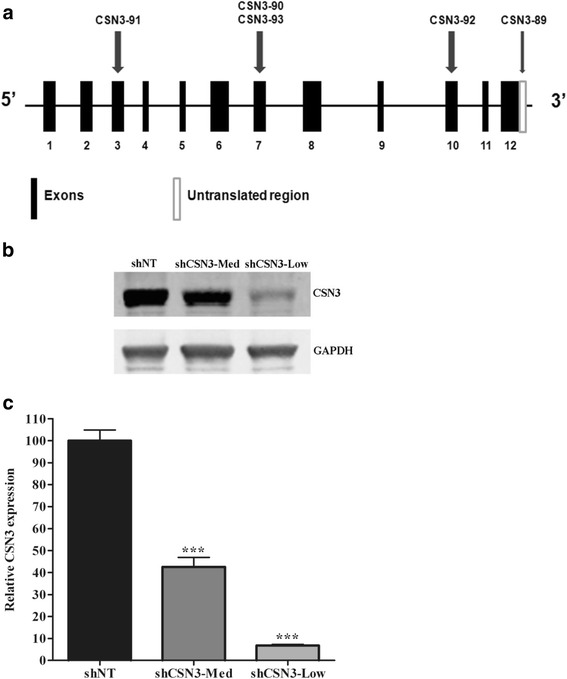
Down regulation of CSN3 in C2C12 cell lines. **a** Representation of the CSN3 gene with arrows indicating the shRNAs target regions. **b** Low passage C2C12 were infected with lentiviral vectors expressing shCSN3-Med, shCSN3-Low or non-target shRNA (shNT). Stable cells lines were selected with puromycin (1.5 μg/ml). Total protein (20 μg) was analyzed by immunoblots using CSN3 and GAPDH (internal control) antibodies. A representative blot is shown from samples separated on a single gel. **c** CSN3 expression was quantified and normalized to GAPDH. Data represent means ± SEM for 7–8 independent samples. Data were analyzed by one-way ANOVA, ***P <0.001 compared to shNT-control

To generate CSN3 stable knockdowns, we first tested 5 distinct shRNAs targeting the CSN3 gene. As shown in Fig. 1a, shCSN3-89 targets the 3’untranslated region (UTR), shCSN3-90 and shCSN3-93 target exon 7, shCSN3-91 binds to exon 3, and shCSN3-92 targets exon 10 (Fig. 1a). Stable cell lines expressing the CSN3 shRNAs produced different degrees of CSN3 knockdown relative to those expressing the shNT viral control. The shCSN3-89 stable cell line showed the lowest (shCSN3-Low) expression of CSN3 protein (7%) and shCSN3-90 produced a mid-level (shCSN3-Med) expression of CSN3 protein (43%) relative to shNT-control cells (Fig. 1b-c). shCSN3-Low and shCSN3-Med stable cell lines are referred to as “CSN3 knockdowns.” All subsequent experiments were completed using these stable knockdowns. Their level of CSN3 expression remained stable throughout the study period.

### Knockdown of CSN3 reduces the stability of other CSN complex subunits

**Fig. 2:**
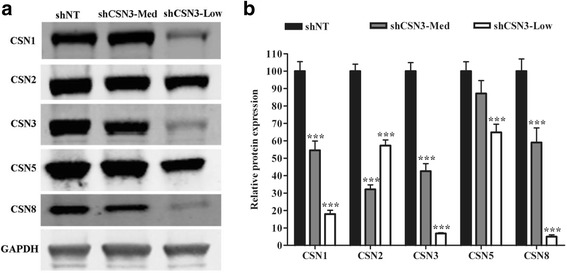
Knockdown of CSN3 decreases the protein levels of other CSN subunits **a** Proteins were extracted from proliferating shNT-control, shCSN3-Med or shCSN3-low stable cells lines. Total protein (20 μg) was separated by SDS-PAGE, transferred to nitrocellulose membranes, and probed for antibodies against CSN1, CSN2, CSN3, CSN5, CSN8 or GAPDH. Representative blots are shown for each antibody from samples run on a single gel. **b** The protein expression of each subunit was quantified and normalized to GAPDH. For each CSN subunit, the average protein intensity in shCSN3-Med and CSN3-Low cell lines was normalized to shNT-control cells set at 100. Data represent means ± SEM for at least 6 independent samples for each cell line. Data were analyzed by two-way ANOVA, ***P <0.001 compared to shNT-control

The CSN complex is composed of 8 subunits (CSN1-CSN8). Others have shown that knockdown of CSN1 and CSN3 in Hela cells was accompanied by proportional reduction of the CSN complex, whereas knockdown of CSN5 in the same cell line did not have any impact on the complex [30, 31]. These findings highlight a crucial role for CSN1 and CSN3 in the stability of CSN complex. To determine the effect of CSN3 knockdown on other CSN subunits in skeletal muscle, we performed immunoblot analysis on cells lysates from shNT-control, shCSN3-Low or shCSN3-Med stable cell lines. The lysates were probed for CSN1, CSN2, CSN3, CSN5 or CSN8 expression (Fig. 2). The results show that differential expression of CSN3 in shNT-control, shCSN3-Low and shCSN3-Med is accompanied by a proportional decrease in CSN1, CSN5 and CSN8 protein. The decrease in CSN5 expression was relatively smaller (Fig. 2) and the decrease in CSN2 was not proportional to CSN3 expression. Overall, these results are consistent with previous studies in other cell types [2, 32, 33]. Therefore, the dramatic decrease in both CSN1 and CSN8 subunits indicates that CSN3 is likely required for the stability of the CSN complex in skeletal myoblasts.

### CSN3 Knockdown inhibits myoblast fusion

**Fig. 3:**
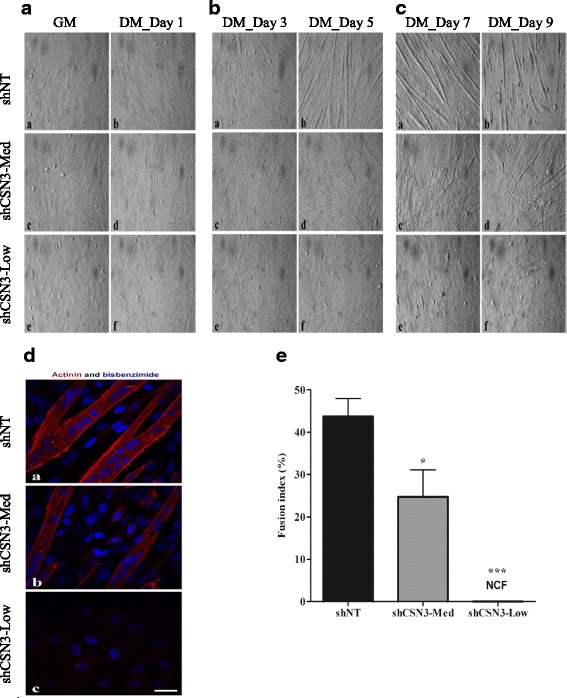
Effect of CSN3 Knockdown on myoblast fusion. (**a**, **b**, **c**) Phase contrast images (20X objective) of stable cell lines grown in differentiation (DM) media from 0 to 9 days. (panels a-b) shNT-control, (panels c-d) shCSN3-Med and (panels e-f) shCSN3-Low cells. Cells were seeded in growth media (GM) until 70–80% confluent (**a**, panels a, c, e) and induced to differentiate for up to 9 days. (**d**) Immunofluorescence images. Cells were grown for 5 days in DM, fixed and stained with anti sarcomeric α actinin (red) and bisbenzimide (blue). Images were acquired with a confocal microscope (60X objective). Scale bar = 30 μm. (**e**) Index of fusion representative quantification. No cell fusion (NCF), *** *P* <0.001 significantly different from shNT-control

Our previous work showed that CSN3 is a newly identified downstream effector of β1D integrin [19]. The β1D integrin isoform is only expressed in mature striated muscle, localizes to focal adhesions during muscle cell differentiation and its knockdown impairs myogenesis [19, 34, 35]. However, the role of CSN3 in skeletal muscle myogenesis remains unclear. To determine the role of CSN3 in skeletal muscle cells differentiation, we investigated if a decrease in CSN3 affects myoblast fusion during skeletal muscle differentiation. The three stable cells lines (shNT-control, shCSN3-Low and shCSN3-Med) were cultured in GM until they reached 70–80% confluency (Fig. 3a, panels *a*, *c* and *e*). Then, GM was replaced with differentiation media (DM) and myotube formation was monitored up to 9 days (Fig. 3). After 1 day in DM, shNT-control and shCSN3-Med cells were elongated, while shCSN3-Low cells did not show any morphological changes (Fig. 3a, panels *b*, *d* and *f*). In contrast to knockdown cells, after 3 days in DM, shNT-control cells began to fuse into myotubes (Fig. 3b, panels *a*, *c* and *e*). After 5 days in DM, the shNT-control myotubes were thick elongated myotubes, shCSN3-Med cells were shorter and thinner myotubes indicating a lack of normal hypertrophy and shCSN3-Low cells appeared unchanged (Fig. 3b, panels *b*, *d* and *f*). In contrast to shNT-control myotubes, even after 9 days, shCSN3-Med myotubes did not hypertrophy (remained thin, Fig. 3c). To illustrate myoblast differentiation, on day 5 cells were fixed and stained for nuclei and sarcomeric α-actinin (Fig 3d). As shown, shNT-control cells had sarcomeric α-actinin at Z-bands indicative of differentiation (Fig 3d, panel *a*). Similarly, shCSN3-Med cells had sarcomeric α-actinin at Z-bands, albeit at reduced levels (Fig 3d, panel *b*). Finally, shCSN3-Low cells did not express sarcomeric α-actinin (Fig. 3d, panel c). To quantitate the extent of myoblast fusion, the fusion index was calculated as the percent of sarcomeric α actinin positive myotubes with three or more nuclei divided by the total number of nuclei per field (Fig. 3e). The fusion index of shNT-control was significantly higher (43% ± 4.23) than shCSN3-Med (24% ± 6.23) or CSN3-low, which did not show any cell fusion (0%). These results demonstrate that knockdown of CSN3 inhibits myoblast fusion and maturation in a dose-dependent manner.

### CSN3 knockdown alters the expression of muscle differentiation markers

**Fig. 4:**
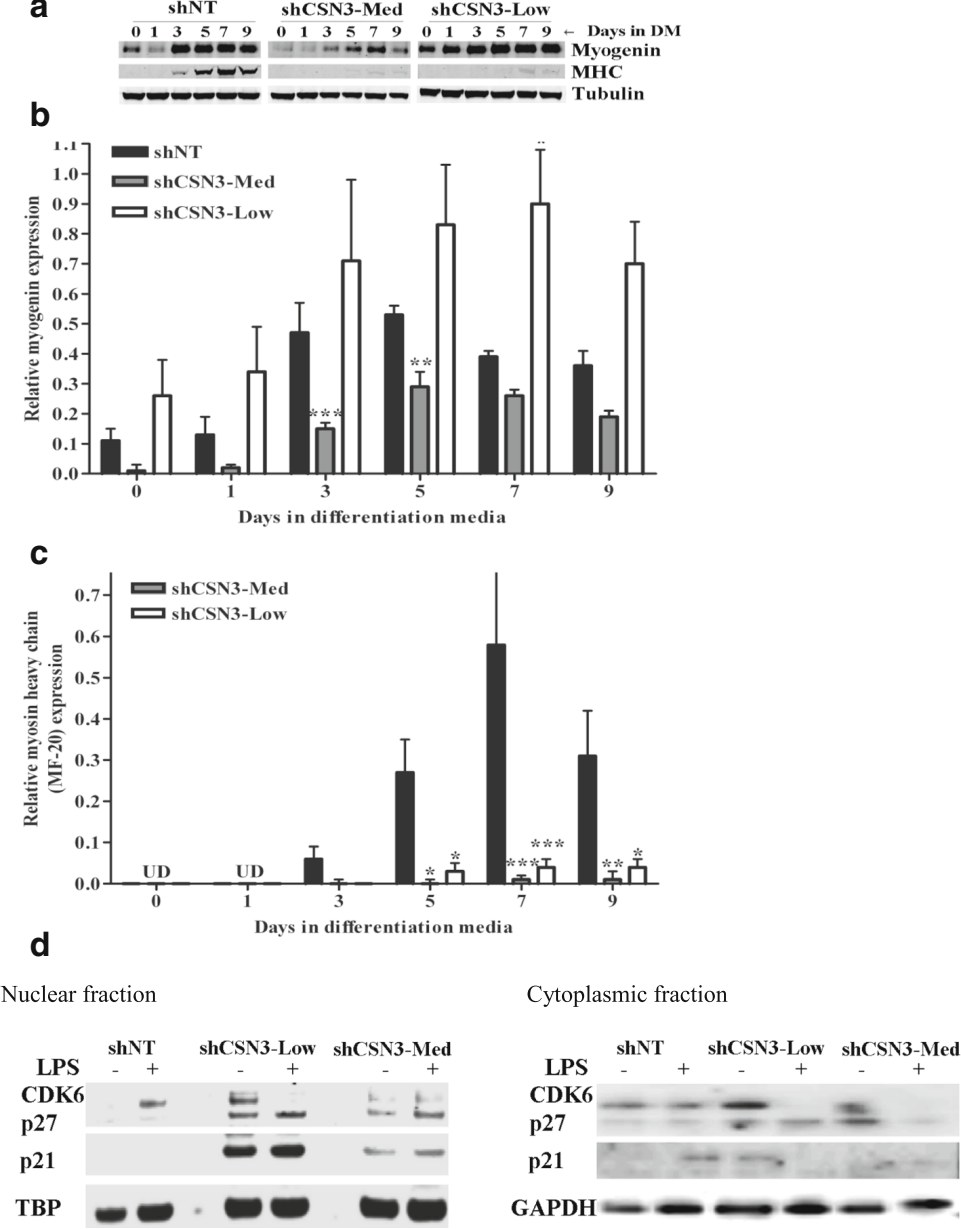
Effect of CSN3 knockdown on the expression of myogenic markers. **a** Representative immunoblots of myogenin and MHC expression in C2C12 cell lines from samples run on a single gel. **b** A histogram showing the quantification of myogenin expression normalized to tubulin. **c** Quantification of MHC normalized to tubulin. The shNT-control, shCSN3-Med and shCSN3-Low stable cell lines were cultured in growth media until 70–80% confluence (0 day) and switched to differentiation media (DM) from 1 to 9 days. Total protein (18 μg) was analyzed by immunoblot using myogenin and MHC antibodies. All blots were reprobed for tubulin (internal control). UD, undetectable. Values represent a mean of 4 independent experiments ± SEM. *p <0.05, **p <0.01 and ***p <0.001 versus time matched shNT-control, by two-way ANOVA. **d** Representative immunoblots of CDK6, p21 and p27 nuclear fractions (*left*) and cytoplasmic fractions (*right*) from samples run on a single gel. Cells treated with 1 μg/ml lipopolysaccharide (LPS) were used as internal controls to verify immunoreactive bands

Myoblast fusion is preceded by the expression of early myogenic markers, such as myogenin and is followed by the expression of muscle specific genes, such as sarcomeric MHC (MHC). To test the effects of CSN3 knockdown on the expression of these muscle differentiation markers, protein obtained from cells following 0 to 9 days in DM, was analyzed by immunoblotting using antibodies against myogenin and MHC to show the progression of differentiation (Fig. 4). Myogenin was detected and showed to increase in a pattern indicative of differentiation in shCSN3-Med and shNT-control lysates. As expected, myogenin expression was significantly lower in shCSN3-Med lysates at days 3–5 of differentiation when compared to the shNT-control lysates. However, high levels of myogenin were found in shCSN3-Low lysates at all time points (Fig. 4a-b). MHC expression was not detected in shNT-control cells at 0 or 1 days, but was up regulated thereafter (Fig. 4a–c). In contrast, MHC expression in the CSN3 knockdowns was significantly lower than the shNT control lysates up to 9 days post-differentiation (Fig. 4a–c).

Cell cycle withdrawal, prior to myoblast fusion, is a required step in muscle cell differentiation. CIP/KIP family proteins, including p21 and p27 are crucial mediators of the cell cycle withdrawal necessary for the onset of differentiation [36, 37]. p21/Cip1, p27/Kip1 proteins form complexes with cyclins to inhibit kinase activity, and consequently block progression of the cell cycle through G1/S. CDK6 is a member of Cyclin-dependent kinase (CDK/Cyclins) family, heterodimer serine/threonine protein kinases involved in cell cycle progression and differentiation [36, 38]. CDK6 has been shown to stimulate cell-cycle progression and a decrease in CDK6 is necessary for terminal differentiation of several cell types [37]. To investigate the role of CSN3 in cell cycle arrest, cells were grown to confluency, fed with differentiation media for 48 h and then lysates were immunobloted with p21/Cip1, p27/Kip1 and CDK6 antibodies. An additional group of cells were treated with 1 μg/ml LPS as a known effector of cell cycle progression to verify immunoreactive bands. Knockdown of CSN3 correlated with significantly increased levels of nuclear p21/Cip1, p27/Kip1 and CDK6 in growth arrested shCSN3-low cells, and to a smaller degree in shCSN3-Med cells, relative to the control cells (Fig. 4d). The increased level of p21/Cip1 and p27/Kip1 proteins is indicative of cell-cycle arrest at G1/S under starvation conditions. However, the increased expression of CDK6 may inhibit differentiation/fusion of the myoblasts under conditions that would normally stimulate differentiation. The stoichiometric balance of these proteins could ultimately dictate the fate of the myoblasts.

### Knockdown of CSN3 impairs proliferation of C2C12

**Fig. 5:**
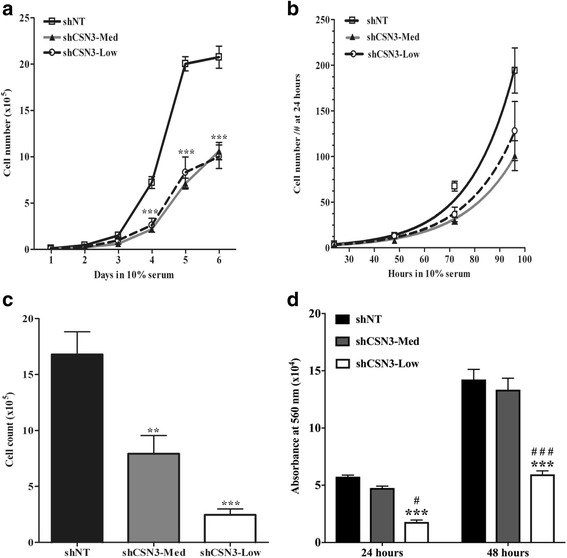
Knockdown of CSN3 inhibits myoblasts proliferation. shNT-control, shCSN3-Med and shCSN3-Low cells were seeded at the same density, serum-starved for 48 h, and treated with growth media containing 10% FBS for the indicated time points (1 to 6 days). Cells were trypsinized and counted using a Coulter counter or by flow cytometry. MTT assay was performed on a 96-well plate 24 or 48 h post proliferation. **a** Growth curve generated from cell count obtained by Coulter counting. **b** Best-fit curve of the doubling times from 6 experiments, standardized to 24 h for each cell type, were significantly different (*p* < 0.0002). **c** Cells were counted by flow cytometry 48 h post growth. **d** MTT assay 24 or 48 h post proliferation. Values represent means ± SEM of 6 (Fig. 5a-b) and 4 (Fig. 5c) independent experiments, ** *** and #, # # # significantly different from the time matched shNT-control or from shCSN3-Med as determined by one-way (**c**) or two-way (**a**) ANOVA. **p <0.01 and ***p <0.001

Previous work has demonstrated the involvement of CSN in cell cycle regulation and in cell proliferation [39, 40]. However, the effect of CSN3 on C2C12 myoblast proliferation is unknown. To investigate this, cell proliferation for CSN3 knockdown myoblasts were evaluated with three independent methods. First, cells plated at low density were collected and counted with the coulter counter over 6 days. The resultant growth curves were plotted and are shown in Fig. 5a. The growth curves showed that shNT-control, shCSN3-Med and shCSN3-Low cell lines had a lag phase of 3 days. However, exponential growth rates between days 3 and 5 were significantly slower for shCSN3-Med and shCSN3-Low cells relative to shNT-control cells. By day 6, the shCSN3-Med cell line did not appear to be entering stationary phase while both shCSN3-Low and shNT-control cells did. To precisely compare their proliferation rates, their doubling time was evaluated based on the exponential growth equation and a best-fit curve was generated using Graphpad Prism software. Doubling time was significantly slower (*p* <0.0002) in CSN3 knockdowns than in shNT-control cells (Fig. 5b). Second, to independently confirm these differences, cell proliferation was measured using both flow cytometry and an MTT assay. Equivalent numbers of exponentially growing cells were serum-starved for 48 h, and then allowed to grow for 24 or 48 h. Following collection, flow cytometry was used to determine cell counts. As shown, the shCSN3-Med and shCSN3-Low cell counts were significantly lower than shNT-control cells (Fig. 5c). The MTT assay reveals that shCSN3-Low cell numbers were significantly lower than shCSN3-Med and shNT-control cells throughout the proliferation period (Fig. 5d). Interestingly, 24 h post-proliferation, we note that both CSN3 knockdown lines display reduced absorbance compared to shNT controls (Fig. 5d). These results suggest that CSN3 is required for the proliferation of C2C12 myoblasts.

### CSN3 Knockdown alters cell cycle progression

**Fig. 6:**
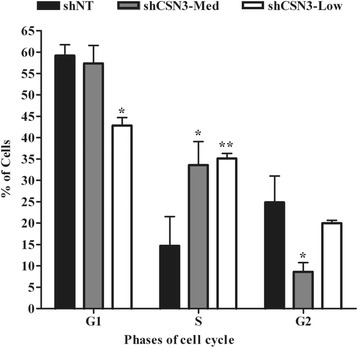
Impact of CSN3 knockdown on cell cycle progression. shNT-control, shCSN3-Med and shCSN3-Low cells were seeded at equal densities, serum-starved for 48 h, and treated with growth media containing 10% FBS for 48 h. Cells were trypsinized, stained with propidium iodide and the cell cycle was analyzed by flow cytometry. Values represent averages ± SEM of 3 independent experiments, * ** significantly different from shNT-control as analyzed by two-way ANOVA. *p <0.05 and **p <0.01

Given the aberrant proliferation of CSN3 knockdowns, cell cycle progression was examined. To evaluate the cell cycle checkpoints, shCSN3-Med, shCSN3-low and shNT-control cells in exponential phase were stained with propidium iodide (PI) and analyzed by flow cytometry. The cell cycle distribution of shNT-control cells was 59% ± 2.54 in G1 phase, 14% ± 6.85 in S phase and 24% ± 6.17 in G2-phase. The population of cells in S-phase for the knockdowns was double the control (~32%). Additionally, shCSN3-Low exhibited a significant decrease in G1 (42% ± 1.88) and shCSN3-Med in G2 (8% ± 2.16) compared with shNT control (Fig. 6). Interestingly, CSN3-Low displayed a significant decrease in G1 phase cells compared with the CSN3-Med and shNT-control (Fig. 6). Overall, these results indicate that knockdown of CSN3 delays the S-to-G2 progression.

### CSN3 signals through NF-κB to regulate C2C12 Differentiation

**Fig. 7:**
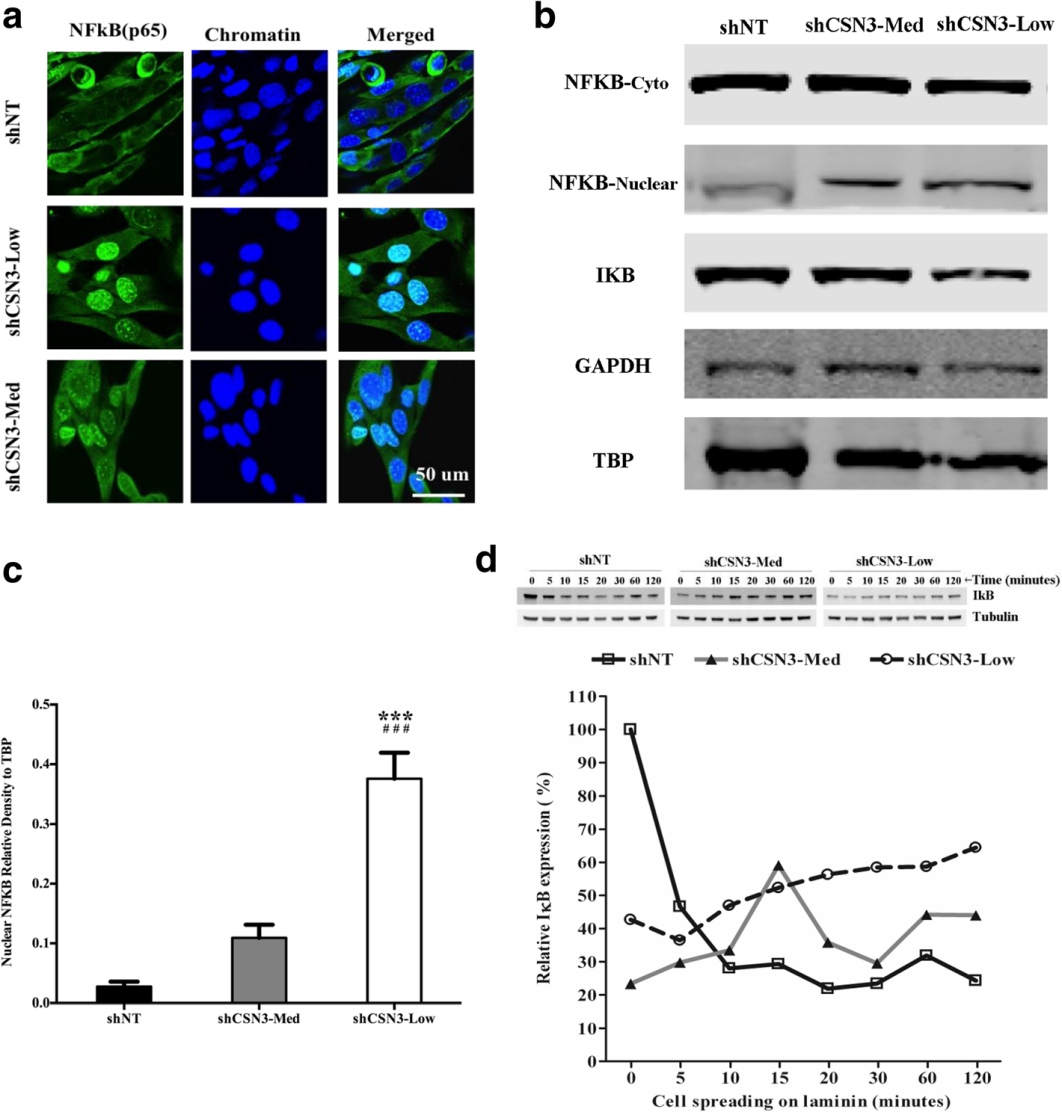
Down regulation of CSN3 alters NF-κB-mediated signaling. **a** Immunofluorescence images of cells labeled with NF-κB p65 (*Green*) and Dapi (*Blue*). **b** The shNT-control and CSN3 knockdowns were grown to confluency, and differentiated for 48 h. Nuclear and cytoplasmic fractions were obtained and analyzed by immunoblot. **c** Quantification of nuclear NF-κB p65 expression normalized to TATA binding protein (*TBP*). * ** significantly different from shNT-control, # # # significantly deviated from shCSN3-Med group as analyzed by two-way ANOVA. *** and # # # *p* <0.001. **d** Quantification of IκB expression normalized to tubulin. Representative immunoblots are shown from samples run on a single gel for each antibody

Nuclear factor κB (NF-κB) is a transcription factor that mediates a plethora of cellular processes such as inflammation, cell growth and differentiation. CSN3 has been shown to bind to kinases (IKKγ, CK2 and PKD) known to phosphorylate IκB (biological repressor) and c-Jun. Phosphorylation of IκB results in its dissociation from NF-κB, its ubiquitination and subsequent degradation by the 26S proteasome complex, and consequently to NF-κB translocation into the nucleus where it controls gene transcription [41–43]. To test whether CSN3 signals through the NF-κB pathway, confluent cells were serum-starved to induce differentiation. After 48 h, cells were examined for morphological changes. As shown, shNT-control cells were fused into myotubes (Fig. 7a). Similarly, shCSN3-Med began to differentiate, albeit to a lesser extent relative to the shNT-control and shCSN3-low did not appear to initiate differentiation. The degree of differentiation correlated with alterations in the amount of nuclear NF-κB observed, and was inversely correlated with IκB for shCSN3-Low (Fig.7b). Specifically, shCSN3-Low nuclei had high levels of NF-κB, shCSN3-Med had intermediate levels and the control had very little nuclear NF-κB (7C). To further assess NF-κB pathway activation, we analyzed the cells for changes in IκB in response to laminin binding. Prior to plating on laminin, a matrix that promotes myoblast differentiation, shNT-control cells had high levels of IκB whereas the knockdown cells had low levels of IκB. Following binding, the level of IκB in the control cells decreased to the level of IκB found in the knockdown cells and no additional decrease in IκB was seen in the knockdown cells (Fig. 7d). The lack of IκB in the knockdown cells prior to being grown on laminin correlates with their increased nuclear NF-κB and indicates that NF-κB may be activated at baseline in CSN3 knockdown cells.

## Discussion

This study examined the role of CSN3 in skeletal muscle differentiation and proliferation. CSN3 is subunit 3 of CSN complex, a highly conserved multifunctional complex composed of 8 subunits (CSN1-CSN8) present in several organisms [2, 8, 44]. The mammalian CSN complex is involved in numerous molecular and cellular processes including protein degradation and phosphorylation, gene transcription, cell cycle regulation, subcellular localization, apoptosis and development [39, 45–47]. Some of the individual subunits were shown to have an additional role(s) independent of the complex [48]. Tissue specific roles of individual CSN subunits are not well characterized. We previously reported that CSN3 binds to β1D integrin, an isoform of β1 integrin expressed in differentiated cardiac and skeletal muscle. In fact, CSN3 localized to integrin adhesions during muscle cell differentiation in C2C12 cells and Z-bands isolated from adult mice [19]. This work is the first study to examine the role of CSN3 in skeletal muscle proliferation and differentiation.

In this study, we used C2C12 cells as a model cell line to study skeletal muscle differentiation in vitro. These cells are a well-established primary murine myoblast model cell line consistently used for these types of studies [29]. We generated two myoblast cells lines with decreased levels of CSN3 shCSN3-Low and shCSN3-Med. Despite high levels of myogenin expression, the shCSN3-Low cells remain mononucleated up to 5 days in differentiation media. The shCSN3-Med cells had significantly lower levels of myogenin expression relative to control cells but only formed short myotubes. After differentiation for 9 days, neither cell line expressed a significant amount of MHC indicating failure to terminally differentiate. Our results clearly demonstrate that CSN3/CSN knockdown inhibits skeletal muscle differentiation and maturation.

Our study found that CSN3 knockdown in C2C12 led to defective cell proliferation. This is in agreement with the data of others who found that knockout of CSN2, 3, 5 or 8 in mice caused early embryonic death due to compromised cell proliferation and survival [17, 49–51]. CSN is implicated in various phases of the cell cycle. For instance, microinjection of purified CSN complex into cells in the G1 phase inhibits their progression to the S phase [52]. The knockout of CSN8 blocks the reentry of T cells into the cell cycle from the G0 quiescent stage [51]. However, knockout of CSN5 in the same cell type does not influence the G1 phase but blocks progression through S phase [53]. These findings indicated that each CSN subunit has a specific role in controlling the cell cycle progression and may be cell type specific. We found that knockdown of CSN3 in C2C12 blocks cell cycle progression through S phase, which is in contrast from the work of others who showed that knockdown of CSN3 in hepatocellular carcinoma cells led to cell cycle arrest at (G0/G1) [54]. However, both of these results are inconsistent with a previous report, which showed that the knockdown of CSN3 promoted the proliferation of mouse fibroblasts. Together, these contradictory results lend support to cell type specific effects of the CSN3 [40]. Our results confirm the crucial involvement of CSN3/CSN in cell proliferation, and indicate that CSN holo-complex subunits can regulate cell cycle progression at different stages in a cell-type-dependent manner. Overall, shCSN3-Low cells showed impaired proliferation and differentiation accompanied by increased myogenin synthesis. Myogenin is a marker of cell entry into differentiation phase and its expression precedes the postmitotic state, indicating that myogenin expressing cells are capable of dividing [21]. This supports the hypothesis that a significant decrease in the proliferation rate of shCSN3-Low leads to temporary cell cycle arrest causing myogenin synthesis and eventual accumulation in cells that are incapable of undergoing fusion.

To further explore the role of CSN3 and the cell-cycle regulation necessary for myoblast differentiation, we examined the expression levels of p21/Cip1, p27/Kip1 and CDK6 at the onset of myoblast differentiation. The proteins p21/Cip1 and p27/Kip1 are cyclin/CDK inhibitors and are the principal negative regulators of the G1 to S phase transition [55]. The level of all three proteins, p21, p27 and CDK6 were increased in the nuclear fraction of CSN3-Low cells at a very early stage of differentiation. This supports the argument that knockdown of CSN3 may alter the cell-cycle via an increase of nuclear p21 and p27. Additionally, the increased levels of CDK6 may play a role in the inhibition of myoblast-myoblast fusion observed in the shCSN3-Low cultures.

Other groups have reported a decrease in the CSN holo-enzyme when one subunit is knocked out or knocked down. For example, knockout of CSN3 decreases CSN8 in embryos and knockdown of CSN1 and CSN3 decrease the level of the CSN holo-complex proportionally in HeLa cells [17]. In reverse, the knockdown of other CSN subunits led to a decrease in CSN3. For example, knockdown of CSN8 in HEK293 cells and in hepatocytes led to a dramatic reduction in CSN3 and a reduction of the holo-complex [56, 57]. In this study, our data demonstrates medium to low levels of CSN3 significantly decreased the levels of CSN1, CSN2 and CSN8 relative to control cells. Additionally, when CSN3 levels were very low, there was a significant decrease, albeit less dramatic, in CSN5 expression. Furthermore, expression of CSN3, 5, 6, 7 and 8 have been demonstrated to down regulate p27 [48], this correlates with our results showing higher nuclear levels of p21, p27 and CDK6 in CSN3 knockdowns. These results expanded the work of others by demonstrating that CSN3 is crucial for the integrity of the CSN holo-complex, and may control the abundance of cell cycle modulators in skeletal muscle. Our data also supports the notion that CSN1-2-3 and 8, and CSN4-5-6 and 7 exist as separate mini-CSN complexes that link together to form the holo-complex [58].

Our results showed that knockdown of CSN3 led to accumulation of basal NF-κB in the nucleus accompanied by degradation of IκB in growth arrested myoblasts implying that CSN3 acts as a repressor of NF-κB latent activation. These finding corroborate with the reported role of CSN5 in HEK293 cells [59]. In their study, the authors found that depletion of CSN5 increased NF-κB activity. Our experiment also demonstrated that CSN3 knockdown in cells with the highest NF-κB basal activity failed to differentiate into myotubes. This is in agreement with several reports showing that NF-κB is a negative regulator of myogenesis [60–62]. For instance, the knockout of the NF-κB p65 subunit favors skeletal muscle differentiation both in vivo and in vitro [63]. Additionally, embryonic fibroblasts deprived of NF-κB activity showed compromised proliferation [64]. Overall, these studies confirm that CSN3/CSN controls cellular processes such as proliferation and differentiation via an NF-κB-mediated pathway.

## Conclusions

This is the first study toward understanding the role of the CSN3 in skeletal muscle differentiation and proliferation. Our results show that knockdown of CSN3 in C2C12 cells leads to impaired differentiation, proliferation and cell cycle regulation of myoblasts. We also found that CSN3 was essential for the expression of other CSN subunits in myoblasts. This finding supports the notion that the phenotypes observed in CSN3 knockdown cells might reflect that of the CSN holo-complex which is involved in various cellular processes such as cell cycle regulation and proliferation. We previously showed CSN3 and CSN5 bound to β1D-integrin during myoblast differentiation and that the CSN holo-complex colocalized with β1D-integrin at Z-bands in adult cardiac myocytes. The work of others has demonstrated that CSN5 is a negative regulator of NF-κB and a decrease in CSN5 increases nuclear NF-κB thereby stimulating proliferation. We hypothesize that when the CSN holo-complex is bound to β1D-integrin, CSN5 levels are maintained, NF-κB is negatively regulated and the absence of proliferation allows cell cycle withdrawal and myoblast differentiation. In this paper, the knockdown of CSN3 decreased the amount of CSN5 leading to an increase in nuclear NF-κB. NF-κB is known to inhibit C2C12 cell differentiation. This would compete with the increase in myogenin a marker of the initiation of differentiation. However, we find the majority of cells stuck in the S-G2 transition, which is consistent with the fact, that DNA synthesis is not inhibited in the presence of myogenin. this is inconsistent with the increased expression of p27, which normally slows down proliferation by impairing the G1-S transition of the cell cycle resulting in a decrease in the rate of cell division and allowing cell fusion. We believe the absence of CSN3 and CSN 5 alters the homeostasis of each of these regulatory proteins such that cell proliferation is slowed down but at the same time differentiation is inhibited by increased NF-κB. This study clarifies the first step toward unrevealing the CSN3/CSN-mediated pathways that controls C2C12 differentiation and proliferation.

## Abbreviations

CDK6: Cyclin-dependent kinase 6
CSN: COP9 signalosome
CSN1, CSN2, CSN3, CSN4, CSN5 or jab1, CSN6, CSN7 and CSN8: COP9 signalosome subunits one through eight, respectively
DM: Differentiation media
DMEM: Dulbecco’s Modified Eagle Medium
FBS: Fetal bovine serum
GAPDH: Glyceraldehyde 3-phosphate dehydrogenase
GM: Growth media
ITS: Insulin-transferrin-selenium
MHC: Sarcomeric myosin heavy chain
MPN: Mpr1-Pad1-N-terminal (MPN) domain
MTT: 3-(4,5-Dimethylthiazol-2-yl)-2,5-diphenyltetrazolium bromide
NF-κB: Nuclear factor kappa-light-chain-enhancer of activated B cells
PCI: Proteasome, COP9 signalosome, and an initiation factor 3
PI: Propidium iodide
shNT: Non-target shRNA
shRNA: Small-hairpiRNA
